# Research Progress on Phytopathogenic Fungi and Their Role as Biocontrol Agents

**DOI:** 10.3389/fmicb.2021.670135

**Published:** 2021-05-28

**Authors:** Yan Peng, Shi J. Li, Jun Yan, Yong Tang, Jian P. Cheng, An J. Gao, Xin Yao, Jing J. Ruan, Bing L. Xu

**Affiliations:** ^1^College of Agriculture, Guizhou University, Guiyang, China; ^2^College of Plant Protection, Gansu Agricultural University, Lanzhou, China; ^3^Key Laboratory of Coarse Cereal Processing in Ministry of Agriculture and Rural Affairs, Schools of Food and Biological Engineering, Chengdu University, Chengdu, China

**Keywords:** phytopathogenic fungus, toxin, abiotic stress, biological control, application prospect

## Abstract

Phytopathogenic fungi decrease crop yield and quality and cause huge losses in agricultural production. To prevent the occurrence of crop diseases and insect pests, farmers have to use many synthetic chemical pesticides. The extensive use of these pesticides has resulted in a series of environmental and ecological problems, such as the increase in resistant weed populations, soil compaction, and water pollution, which seriously affect the sustainable development of agriculture. This review discusses the main advances in research on plant-pathogenic fungi in terms of their pathogenic factors such as cell wall-degrading enzymes, toxins, growth regulators, effector proteins, and fungal viruses, as well as their application as biocontrol agents for plant pests, diseases, and weeds. Finally, further studies on plant-pathogenic fungal resources with better biocontrol effects can help find new beneficial microbial resources that can control diseases.

## Introduction

Plant diseases result in an annual estimated loss of 10–15% of the world's major crops, with direct economic losses of up to hundreds of billions of dollars (Chatterjee et al., 2016). Of these diseases, 70–80% are caused by pathogenic fungi. Plant-pathogenic fungi have adverse effects on crop growth and yield (Li et al., 2017). In recent years, fungal diseases of crops have become increasingly serious as they have severely affected crop yield and quality, and they have become an important bottleneck for the development of sustainable agricultural (Marin-Menguiano et al., 2019). Some diseases are not caused by a single pathogen, but rather are the result of the synergy of multiple pathogens (Chatterjee et al., 2016). In the long evolutionary history of plants and pathogenic fungi, highly specialized and extremely complex relationships have formed, creating a pattern of mutual selection and co-evolution. Therefore, with the continuous emergence of new varieties and variations of pathogenic fungi, their relationships with plants have also changed (Quintanilha-Peixoto et al., 2019). Based on agricultural production practices and by combining genetic variation and antifungal disease breeding using biochemical, cell biology, and molecular biology methods, a comprehensive analysis of the mechanism of the interaction between pathogenic fungi and host plants is required. We should focus on studying the identifying characteristics of phytopathogenic fungi and hosts, signal transmission pathways, and the regulation of defense responses (Wang et al., 2019b). The isolation and functional verification of pathogenic fungi effectors will lay a theoretical foundation for the development of new disease control approaches, the effective control of pathogen damage, and the selection of resistant varieties (Yang et al., 2019).

Phytopathogenic toxins are non-enzymatic compounds that are harmful to plants and are produced through the metabolism of plant pathogens (Thynne et al., 2017). Very low concentrations of these toxins can destroy the normal physiological functions of plants. Phytopathogenic fungi produce toxins that can play a key role in the development of plant diseases, thereby adversely affecting the host plants (Soyer et al., 2015). Phytopathogenic toxins are mostly low-molecular-weight secondary metabolites that can produce specific symptoms such as wilting, growth inhibition, chlorosis, necrosis, and leaf spotting (Yin et al., 2016). The mechanism of action of phytopathogenic toxins is complex. It mainly acts on the cell membrane, mitochondria, and chloroplasts of host plants, thereby destroying the plant or interfering with its metabolism (Shang et al., 2016). In addition, it inhibits the synthesis of proteins and nucleic acids in the host plant, resulting in physiological disorders, cell death, and even death of the plant itself (Zeilinger et al., 2016). Studying pathogenic toxins and their mechanisms of pathogenicity is of great significance for understanding the interaction between plant hosts and pathogens, as well as for the use of pathogenic toxins to identify plant disease resistance, to screen for disease-resistant mutants, and to control disease (Hamel et al., 2012; Wirthmueller et al., 2013; Bi et al., 2016; Adam et al., 2018). To date, there have been few reviews of phytopathogenic fungi and their toxins (Shang et al., 2016). In this review, we discuss the pathogenic factors of plant-pathogenic fungi, such as cell wall-degrading enzymes, toxins, growth regulators, effector proteins, and fungal viruses, and their application in the biological control of plant diseases, insect pests, and weeds.

## Pathogenic Factors of Phytopathogenic Fungi

**Figure 1:**
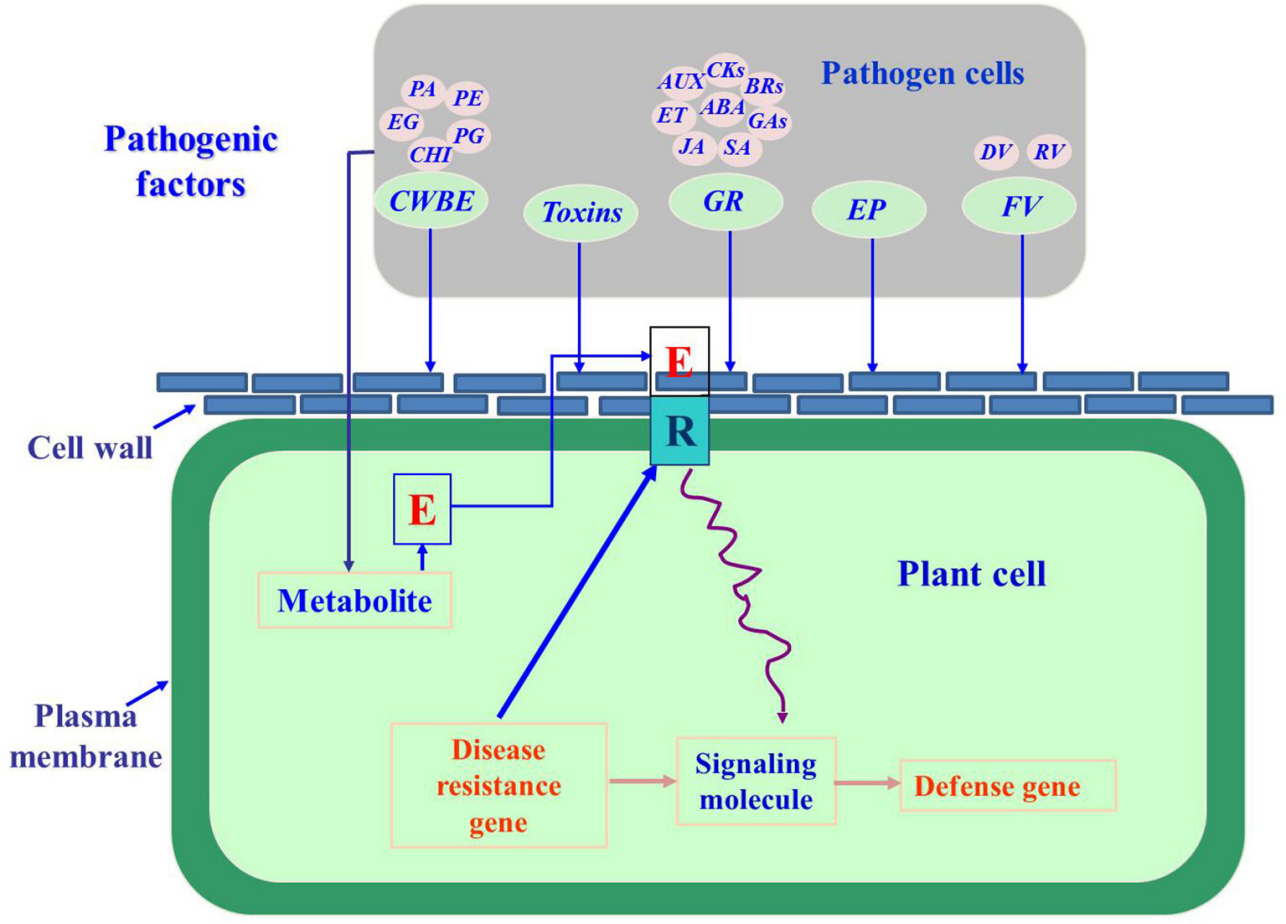
The pathogenic factors of phytopathogenic fungi (Lara-Márquez et al., 2011; Zhang et al., 2014; Gomes et al., 2015; Guerriero et al., 2015; Li et al., 2017; Tan and Oliver, 2017; Proctor et al., 2018; Abro et al., 2019; Azhar et al., 2019; Cobos et al., 2019). CWBE, cell wall-degrading enzymes; PG, pectin polygalacturonase; PE, pectin methylesterase; EG, Endo-1,4-β-D-glucanase; PA, protease; CHI, chitinase; GR, growth regulator; AUX, auxin; CKs, cytokinins; BRs, brassinolides; ABA, abscisic acid; GA, gibberellic acid; SA, salicylic acid; JA, jasmonic acid; ET, ethylene; EP, effector proteins; FV, fungal virus; DV, DNA virus; RV, RNA virus.

Fungal pathogenic factors refer to compounds that are produced when phytopathogenic fungi come into contact with plants. Their metabolism is initiated in fungi by the physiological and biochemical reactions that occur during processes such as surface molecular interactions and signal transmission, and these factors act as pathogens to plants (Félix et al., 2019; Vincent et al., 2020). These factors are mainly enzymes, such as cell wall-degrading enzymes, toxins, growth regulators, and their analogs (Figure 1) (Schiøtt et al., 2010).

### Cell Wall-Degrading Enzymes

Cell wall-degrading enzymes degrade the cell wall and cuticle of the host plant and facilitate the invasion, colonization, and expansion of pathogenic fungi (Zhao et al., 2013; Guerriero et al., 2015). The interaction between cell wall-degrading enzymes of plant-pathogenic fungi and plants during the infection process is gradually being studied through the development of the fields of molecular biology and proteomics. The main cell wall-degrading enzymes are pectinase, chitinase, cellulase, and protease (Lara-Márquez et al., 2011). Guerriero et al. (2015) found that cell wall-degrading enzymes produced by *Rhizoctonia solani* have an obvious degradation effect on the maize radicle, and the degradation ability significantly increases with increasing cell wall-degrading enzyme concentration. In living plant tissues, cellulases secreted by pathogens play a role in the softening and decomposition of cell wall material (Lara-Márquez et al., 2011; Janusz et al., 2017). *Fusarium graminearum* secretes cell wall-degrading enzymes such as cellulase, xylanase, and pectinase during the infection and expansion process, causing the decomposition of host cell wall components and cell wall relaxation, which are beneficial for the penetration and expansion of the pathogen in the host tissue (Beliën et al., 2007). In particular, β-galactosidase is a cell wall-degrading enzyme that can promote the degradation of lactose in the cell wall, which leads to production of galactose and glucose and accelerates fruit softening (Félix et al., 2018). Ma et al. (2019) suggested that β-galactosidase is abundant in the early stages of fruit softening and participates in the degradation of cell wall galactosyl bonds, leading to a decrease in cell wall integrity. Some enzymes, including hemicellulase, protease, amylase, and phospholipase, which can degrade hemicellulose, protein, starch, and lipids, respectively, also play specific roles in the pathogenic process (Félix et al., 2018). Pathogenic fungi infect plants, and disease is caused not only by cell wall-degrading enzymes, but also by hormones, toxins, and other factors. At the same time, pathogen infection can also activate plant defense enzyme systems and induce plants to produce antifungal substances to inhibit the cell wall-degrading enzymes of pathogens and achieve disease resistance. Thus, the infection of plants by pathogenic fungi is a complex biochemical process.

### Toxins

Phytopathogenic fungi produce toxins that can play a key role in the development of plant diseases (Proctor et al., 2018; Yang et al., 2020). As already noted, most phytopathogenic toxins are low-molecular-weight secondary metabolites that can produce specific symptoms such as wilting, growth inhibition, chlorosis, necrosis, and leaf spotting (Jajić et al., 2019). A previous study analyzed the structure of the Crofton-weed toxin (AAC-toxin) in *Alternaria alternata* by spectroscopy and identified it as tenuazonic acid (TeA) (Pusztahelyi et al., 2015). A study of its targets and herbicidal mechanism showed that TeA can inhibit the release of photosynthetic oxygen and reduce the apparent quantum efficiency of the leaves (Kretschmer et al., 2012). Using the modern technologies of rapid chlorophyll fluorescence induction, protein electrophoresis, isotope labeling, and competitive binding, it was found that the toxin completely inhibits electron transfer activity in photosystem II, and its main target is the D1 protein of this photosystem (Santos et al., 2020). Ascaulitoxin aglycone is one of three metabolites with herbicidal activity produced by *Ascochyta caulina*, and it is also a potential herbicide of *Chenopodium album* (Huffaker et al., 2011). Tentoxin, a powerful toxin produced by the plant pathogen *Alternaria alternata*, is a cyclic tetrapeptide compound (Lou et al., 2013) that inhibits chloroplast development. Phenotypically, chloroplasts are expressed as chlorotic tissue, but tentoxin does not directly affect chlorophyll synthesis. The diphenyl ether compound cyperin is a phytotoxic metabolite produced by the fungi *Ascochyta cypericola, Phoma sorghina*, and *Preussia fleischhakii*, and it is toxic to weeds such as *Cyperus rotundus* and *Phytolacca americana*. Cyperin inhibits plant enoyl (acyl carrier protein) reductase, which is a target site for the chemical synthesis of trichloroarsenic, a diphenyl ether (Huffaker et al., 2011). Toxins can cause an increase in the permeability of the host plant cell membrane, leakage of cell electrolytes, damage to the membrane system, and disorder of the organism's material metabolism, thereby causing it to lose normal physiological functions and potentially die (Lyu et al., 2015). Toxins can damage the inner membrane of the chloroplasts in host plant leaves, and at the same time, cause the disintegration of the basal lamella and cause the chloroplasts to form vesicles, thereby causing serious poisoning effects or death to the host plants. Toxins acting on the mitochondria of the host plant will cause damage to the mitochondrial membrane structure, swelling of the cristae, vacuolization, reduction of the mitochondrial matrix and cristae number, or even cause the cristae to disappear. The application of plant-pathogenic fungal toxins is still in the stage of laboratory research. The use of bioengineering technologies, such as cell engineering and tissue culturing, is expected to overcome this problem and allow for the rapid development of the practical applications of natural toxins.

### Growth Regulators

When they invade plants, most pathogenic fungi produce plant growth regulators, such as auxin (AUX), cytokinins (CKs), gibberellic acid (GAs), ethylene (ET), abscisic acid (ABA), brassinolides (BRs), jasmonic acid (JA), and salicylic acid (SA), to disturb the levels of the endogenous hormones in plants and weaken the plant's defenses (Jaroszuk-Scise et al., 2019). AUX indole acetic acid (IAA), synthesized by the mycelia and conidia of *Magnaporthe oryzae*, may induce plant growth and weaken plant defenses (Fu et al., 2015; Krause et al., 2015). CKs synthesized by *M. oryzae* can affect rice defense, nutrient distribution, and tolerance to oxidative stress induced by fungal infection, thereby enhancing fungal pathogenicity. Increased GA content in plant cells infected by pathogenic fungi may serve as an activation signal, enhance the carbon pool activity of infected plant cells, and provide nutrition for the infecting fungi (Jaroszuk-Scise et al., 2019). The addition of ABA can promote the growth of *Ceratocystis fimbriata* hyphae to a certain extent and can promote the formation of the appressorium in *M. grisea*. ABA is synthesized by various plant-pathogenic fungi, and *M. oryzae* pathogenicity is closely related to its ABA synthesis (Ding et al., 2016; Lievens et al., 2017). ET can promote the pathogenicity of some fungi and inhibit the pathogenicity of others. A high concentration of ET promotes the growth of *Sclerotinia sclerotiorum* hyphae and promotes the germination and hyphal growth of *A. alternata* spores; however, it also inhibits the hyphal growth of *Botrytis cinerea* and the hyphal growth and formation of fruiting bodies of *Agaricus bisporus*. ET synthesized by plants can increase the expression of pathogenic genes of *Botrytis cinerea* and the pathogenicity of *Alternaria tabacum*, and it can also induce the formation of adherent cells, leading to fruit infection (Jaroszuk-Scise et al., 2019). SA can inhibit spore germination and mycelial growth of *Harpophora maydis*, reduce spore germination and hyphal growth of *Fusarium oxysporum*, and significantly inhibit the growth of *Aspergillus flavus*. *M. oryzae* can synthesize JA derivatives and monooxygenase, which changes the JA balance in the host and inhibits its JA signal, thereby weakening the host's defense and resistance mechanisms (Jaroszuk-Scise et al., 2019). The systematic study of phytopathogenic fungal hormone synthesis pathways and signal transduction pathways may provide more targets for the development of new drugs against phytopathogenic fungi.

### Effector Proteins

**Figure 2:**
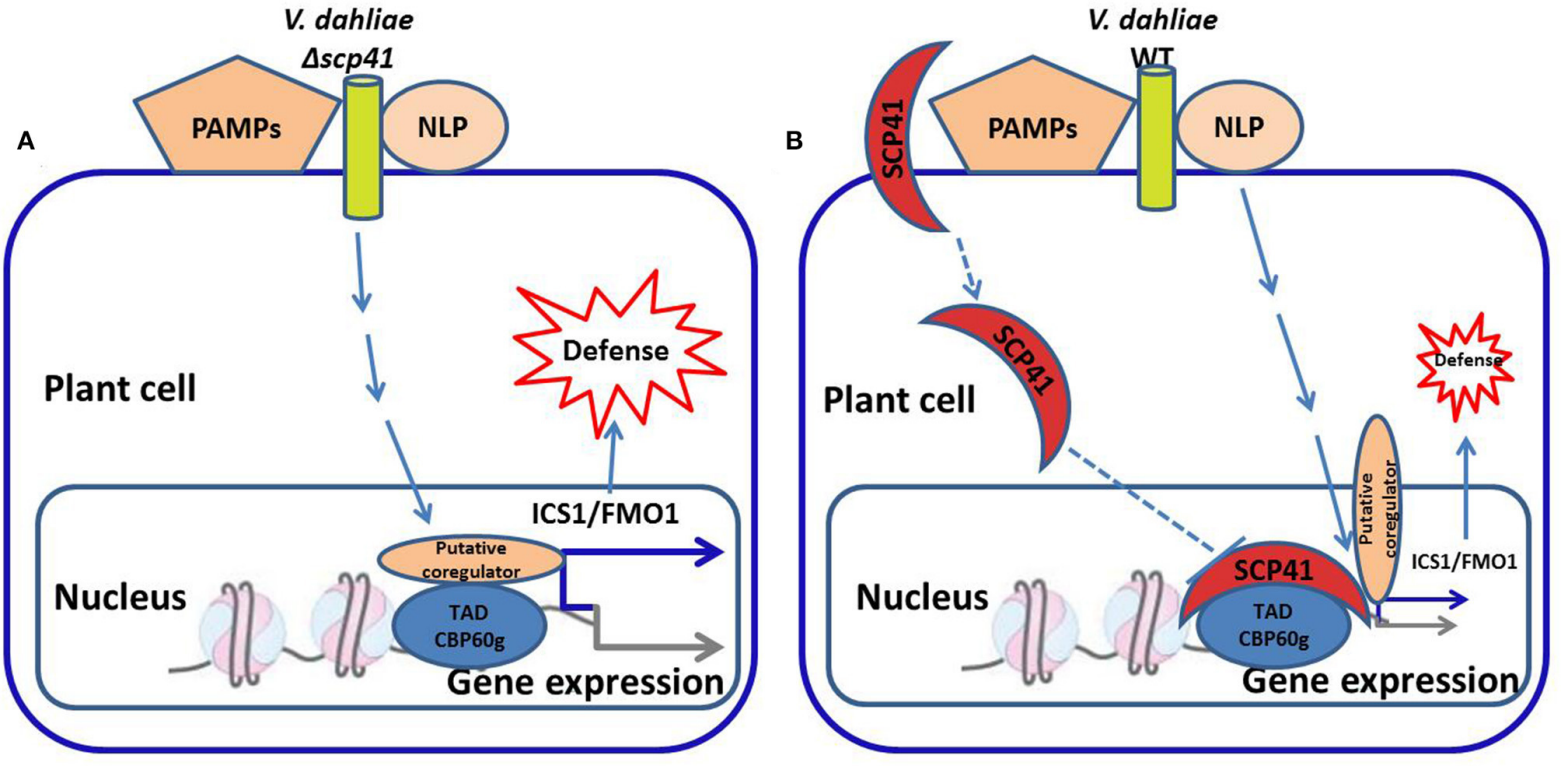
Model for VdSCP41-mediated suppression of Arabidopsis defense during *Verticillium dahliae* infection (Qin et al., 2018; Zhang et al., 2018). **(A)** During the infection of *V. dahliae*, the plant transcription factor CBP60g induces the expression of immune-related genes and the defense response. **(B)** VdSCP41, a *V. dahliae* effector protein, is transported to the nucleus of plant cells and directly targets CBP60g, which interferes with the transcription factor activity and thus suppresses plant immunity. WT, wildtype, PAMPs, pathogen-associated molecular patterns, NLP, nuclear localization protein, SCP41, secretory protein 41, ICS1, isochoric acid synthase 1, FMO1, flavin monooxygenase 1, TAD, transcription activation domain, CBP60g, calmodulin binding protein 60 g.

**Figure 3:**
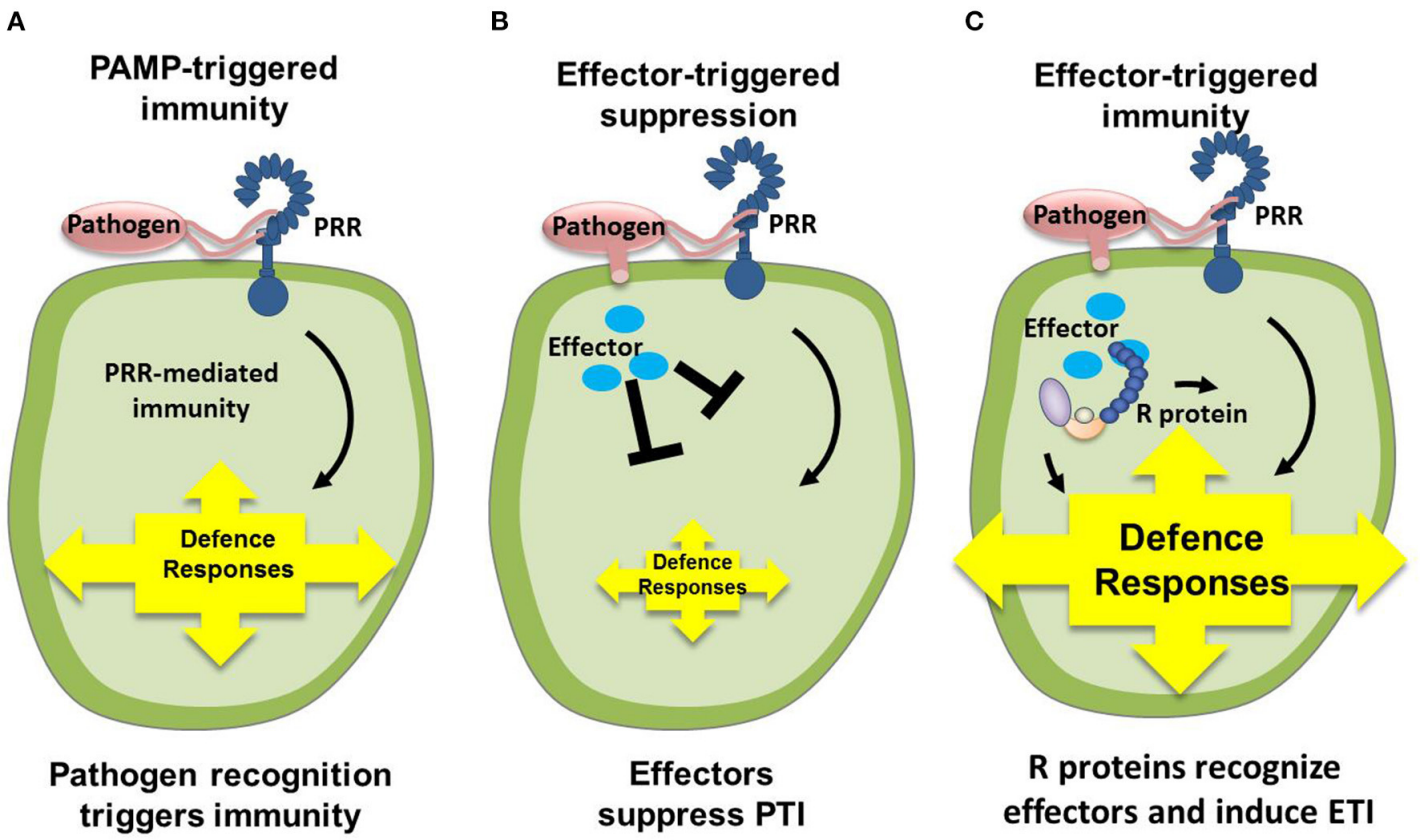
Interaction pattern between phytopathogenic fungi and plants (Pieterse et al., 2009; Cobos et al., 2019). **(A)** Upon pathogen attack, PAMPs activate PRRs in the host, resulting in a downstream signaling cascade that leads to PTI. **(B)** Virulent pathogens have acquired effectors (blue ellipses) that suppress PTI, resulting in ETS. **(C)** In turn, plants have acquired R that recognize these attacker-specific effectors, resulting in a secondary immune response called ETI. PAMP, pathogen-associated molecular patterns; PRR, pattern-recognition receptors; PTI, PAMP-triggered immunity; ETS, effector-triggered susceptibility; R, resistance proteins; ETI, effector-triggered immunity.

Phytopathogenic fungi secrete proteins that can interact with host plants during the process of host infection. These proteins, called effector proteins, play important roles in plant cells and thus affect the interaction between plant pathogens and their hosts. Research on plant-pathogenic fungal effectors has mainly addressed model fungi and those whose whole genome has been sequenced (De Guillen et al., 2015; Tan and Oliver, 2017). To date, the main cloned pathogenic fungal effectors are from *Cladosporium fulvum, Magnaporthe oryzae, Leptosphaeria maculans, Blumeria graminis* f. sp. *hordei, Melampsora lini*, and *Fusarium oxysporum* f. sp. *lycopersici*. *Botrytis cinerea* secretes an exopolysaccharide that can stimulate the SA signaling pathway of the host (Zhang et al., 2019; Tilocca et al., 2020). Due to the antagonistic relationship between SA and JA, the JA signaling pathway of the host is thus inhibited (Guzmán-Guzmán et al., 2017; Liu et al., 2019). The antibiotic biosynthesis monooxygenase produced by *M. oryzae* converts JA to its hydroxyl compound 12OH-JA, which in turn can reduce plant disease resistance (Gomes et al., 2017; Ma et al., 2018; Ökmen et al., 2018). The pathogenic factor VdSCP41 (the *Verticillium dahliae* effector protein) is transported from the fungus to the nucleus of the host plant cell and directly targets the important plant immune regulatory factors CBP60g and SARD1. This process interferes with the host's transcription factor activity, consequently inhibiting the induction of plant immune-related genes and improving the pathogenicity of the fungus (Figure 2) (Lanver et al., 2010; Gomes et al., 2015; Qin et al., 2018). The secreted protein slp1 of *M. grisea* competitively binds to the chitin oligosaccharides that are produced via cell wall degradation. The binding of slp1 prevents the chitin elicitor-binding protein CEBiP in rice from recognizing the chitin oligosaccharides, thus inhibiting the downstream chitin-induced immune response. The effector gene is also called the avirulence gene (*Avr*), and there is a resistance gene (*R*) in the host plant (Wang et al., 2018). The interaction between plant-pathogenic fungi and the host plant can be understood as the interaction between the fungal *Avr* gene and the host plant *R* gene (Qin et al., 2018). Recognition of the *Avr* gene product by the *R* gene product results in incompatibility, such that the plant does not become diseased; when the *R* gene product does not recognize the *Avr* gene product, compatibility with the host plant results in disease (Figure 3) (Kobayashi et al., 2018; Cobos et al., 2019; Yu et al., 2019). The virulence effects and transport molecular mechanisms of effector proteins are still in the initial stages of research. It has not yet been identified which effector proteins can alter plant metabolism to meet the nutritional needs of the infecting fungi, or which plant signal transduction pathways regulate the expression of effector protein genes. Through comparative analysis of more types of fungal effector molecules, common motifs of fungal effectors may be found. Such knowledge would lead to better explanations of the interactions between pathogenic fungi and host plants, and would reveal the pathogenic mechanisms of pathogenic fungi and the disease-resistance mechanisms of host plants.

### Fungal Viruses

Fungal viruses commonly infect various groups of fungi and oomycetes and replicate in their bodies. Most of the reported fungal viruses are RNA viruses, although a few are DNA viruses (Wang et al., 2014; Xiao et al., 2014; Wei et al., 2019). After fungal viruses infect the host fungus, some fungal viruses form a commensal relationship with the host (Lee et al., 2011; Mizutani et al., 2018; Zhai et al., 2019). However, some fungal viruses have a significant negative impact on their fungal hosts (Xiao et al., 2014). This type of virus is often associated with weak virulence of the plant-pathogenic fungus, which leads to a decrease in the pathogenicity of the fungus, specifically by slowing the mycelial growth rate, decreasing spore production, and decreasing pigment content (Hao et al., 2018; Moriyama et al., 2018). In the 1950s, scientists first discovered weak toxicity of plant-pathogenic fungi in *Cryphonectria parasitica*, and they found that low-virulence strains could transmit low virulence to normal strains by means of hyphal fusion (Wu et al., 2012). Torres-Trenas et al. (2019) inoculated an ulcer spot of chestnut blight with a pathogenic fungal strain that had a weakly virulent virus, a slow growth rate, poor sporulation ability, and decreased pigment secretion, and they successfully prevented the disease (Lau et al., 2018; Zhai et al., 2018; Wang et al., 2019a). In the 1990s, the control of chestnut blight with attenuated mycoviruses was very successful in Europe (Zhang et al., 2014; Li et al., 2019). However, the use of similar methods to prevent chestnut blight in the Americas has never achieved the same effect (Liu et al., 2015; Andika et al., 2017; Abdoulaye et al., 2019). This is because the vegetative incompatibility groups of American chestnut blight are more complicated than those in Europe, which hinders the control effect (Wei et al., 2019; Dejasikora et al., 2020). Some phytopathogenic fungi that carry a certain type of virus can exhibit low pathogenicity (virulence decay) characteristics that are mediated by the virus, thereby protecting the host plant (Ding et al., 2019; Thapa and Roossinck, 2019). Azhar et al. (2019) discovered the fungal virulence-attenuating properties of the *Sclerotinia sclerotiorum* strain Ep-IPN and observed that its attenuating factor (dsRNA) could be transferred to a strain with normal vegetative affinity (Ep-IPNA183) through soil and rape leaves, which caused the virulence of the normal strain to decline and protected the plants from any damage caused by the latter strain (Azhar et al., 2019; Torres-Trenas et al., 2020). However, there are many problems that need to be solved when fungal viruses are applied in actual plant production. Factors such as plant cultivation conditions, cultivation measures, the natural and micro-ecological environments, and the morphological stability of fungal viruses all affect their function. Therefore, the use of fungal viruses for disease prevention in the field must consider their ecological, pathological, and morphological effects.

## Application of Pathogenic Fungi For Biological Control

### Biological Control of Insect Pests

**Figure 4:**
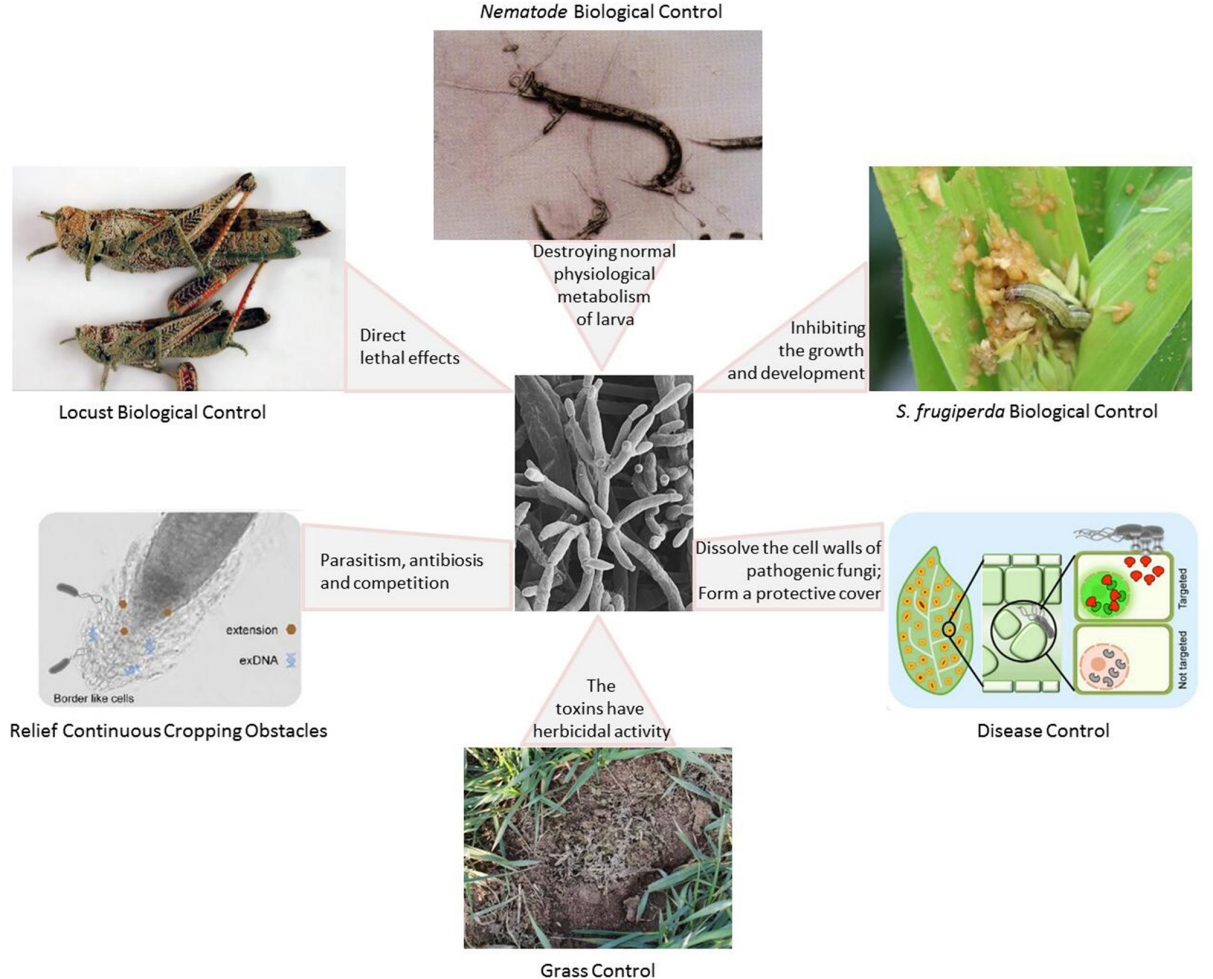
The role of phytopathogenic fungi as a biological control agent with different mechanisms of action (Hamel et al., 2012; Pereira et al., 2014; Clancy et al., 2017; Garrigues et al., 2018; Filizola et al., 2019; Kaur et al., 2019; Moisan et al., 2019; Toju and Tanaka, 2019; Ying and Feng, 2019; Dejasikora et al., 2020).

A large number of studies have shown that the combined use of pathogenic fungi and other biological control methods (including bacteria, plant-derived insecticides, attractants, and natural enemies) can significantly improve the effectiveness of insect-pathogenic fungi (Figure 4) (Zhao et al., 2014; Van den Bosch et al., 2019). Fungus-based insecticides have great potential as a form of pest control. However, the technology bottleneck for large-scale production restricts the large-scale application of fungal pesticides, and it also affects research into and application of fungal pesticide products (Keswani et al., 2014; Bashan et al., 2016). In addition, the safety of phytopathogenic fungi in biological control also requires sufficient attention (Keswani et al., 2014).

#### Locust Biological Control

The biological control of locusts has rapidly developed in recent years with the production of many new technologies and products. Until recently, the biological control of locusts consisted mainly of early applications of natural parasitic enemies and botanical agents (Zhao et al., 2014). Today, the development and application of pathogenic microorganisms that affect locusts is becoming increasingly important. Pathogenic fungi of locusts are mainly distributed in the subclasses *Zygomycetes* and *Deuteromycetes* (Clancy et al., 2018). At present, the locust-toxic pathogenic fungi used in artificial propagation mainly include *Metarhizium anisopliae, Beauveria bassiana*, and *Metarhizium anisopliae*, although *Entomophaga grylli* and *Fusarium verticillioides* are also used (Tralamazza et al., 2019). *M. anisopliae, A. flavus, M. flavoviride*, and *B. bassiana* are commonly used in field demonstrations. These fungi naturally produce fungal spores that can germinate on the surface of the locust and then penetrate its exoskeleton, destroying the locust tissue from the inside. In addition to the direct lethal effects of locust microspores, the sublethal effects, such as inhibition of the growth and development of locusts, egg laying, and gathering behavior, are perhaps more important. Locust microspores can significantly inhibit locust colony migration behavior, mainly by interfering with the synthesis of neurotransmitters, such as serotonin and dopamine, that regulate migratory locust colonies. At the same time, locust microspores can prevent locust aggregation behavior by inhibiting the growth and development of intestinal bacteria involved in locust aggregation pheromone synthesis. Importantly, the microspores do not have a direct toxic effect on vertebrates, therefore reducing the environmental impact on crops and beneficial insects such as bees. There have been a few studies on locust-pathogen subfamilies which have been combined into complexes. The most studied and applied of these is the *E. grylli* complex, which includes at least four pathogenic fungi: *E. calopteni, E. macleodii, E. praxibuli*, and *E. asiatica. Metarhizium acridum*, which specializes in parasitizing locusts, secretes a series of proteins, including proteases, chitinases, and cytochrome P450 (Zhao et al., 2014; Clancy et al., 2018). These proteins enhance the ability of *M. acridum* to adapt to different conditions (Moisan et al., 2019). Biocontrol workers have also tried to add incidental substances, such as neem seed oil, to fungal pesticides to develop new formulations with increased lethality to locusts (Clancy et al., 2017; Shi et al., 2019). After years of follow-up investigation, scientists have found that the natural prevalence rate of fungal diseases in locust swarms is very low, and the natural susceptibility rate in African grassland locust swarms is about 2–6%. Therefore, to control locusts below the economic damage level, artificial propagation and application to locust swarms are necessary to increase the susceptibility rate of locusts and reduce their population density. In future studies, genetic improvement of pathogenic fungal strains may be used to improve strain virulence, universality, and insecticidal speed. We can reduce the cost of fungal pesticide products by improving the fermentation process, as well as by improving the insecticidal effect in the field through better formulation technology.

#### *Spodoptera frugiperda* Biological Control

*S. frugiperda* is a major agricultural pest that is harmful and difficult to control worldwide (Wang et al., 2019c). More than 60% of insect deaths in nature are caused by fungal diseases, and consequently there have been many reports of fungal strains with good control potential for *S. frugiperda* (Clancy et al., 2017). In India, a field investigation found a large number of *S. frugiperda* infected by *Metarhizium riley* (Keswani et al., 2019). Gómez et al. (2018) found that one of the 97 isolates of *Beauveria bassiana* in Mexico is 97% lethal to the second instar larvae of *S. frugiperda*. During the infection process of pathogenic fungi, natural factors such as wind, rain, and the frequency of contact between insects and fungi are important factors that affect the pathogenesis of the host. Carneiro et al. (2008) found that four out of 24 *B. bassiana* strains isolated from Brazil could kill 100% of 2-year-old *S. frugiperda* larvae; the mortality rate of *S. frugiperda* was as high as 100% on the seventh day after inoculation of the third instar larvae of *S. frugiperda* with the *Metarhizium rileyi* strain ZYSP190701 at a concentration of 1 × 10^8^ conidia/mL. The insecticidal rate and spectrum of pathogenic fungi are mainly affected by the fungal infection mechanism, the host insect's defense mechanism, and environmental conditions. Although the insecticidal fungal resources that can infest *S. frugiperda* are abundant in nature, most of them only have good control effects on the young larvae of this pest. There have only been a few reports of fungal strains that are pathogenic to *S. frugiperda* larvae older than 3 years and at other developmental stages (Moisan et al., 2019). The poor control effects of most pathogenic fungi on older *S. frugiperda* larvae may be closely related to the infection mechanism of the fungus and the defense mechanism of *S. frugiperda* (Gómez et al., 2018). For the same strain, there is no significant difference in pathogenicity after continuous culture on artificial culture media or live insects. There may be great differences in the mechanisms of *S. frugiperda* infection by different pathogenic fungi and in the immune response mechanisms of *S. frugiperda*. The virulence of pathogenic fungi is mainly affected by their virulence-related genes and host resistance. Through natural selection and molecular improvement, the breeding of strains that have significant control effects on older *S. frugiperda* larvae and various other insect stages is expected (Wang et al., 2019c). The lack of research on the effects of fungal insecticides on the different developmental stages of *S. frugiperda* has severely restricted the development of fungal insecticide products and the application of technological research on fungal control of *S. frugiperda*. However, specific combinations of insecticides can be used to prevent and control *S. frugiperda*. The combination of chemical insecticides and insect-pathogenic fungi can improve fungal infectivity, while reducing the field dose of chemical insecticides and reducing their negative impact on the environment. In order to fully understand the role of fungal insecticides in the continuous control of pests and reduce the use of chemical pesticides, it is necessary to intensify the research on applied technology based on the selection of insecticidal strains that act on different insect states.

#### Inhibition of Other Pests

Kandasamy et al. (2016) selected 15 phytopathogenic fungi with good spore production and used the spray method to determine their differential pathogenicity to adults and nymphs of the pear stink bug. Strain Bb2359 showed high levels of virulence against adults and nymphs of the pear stink bug, with a 50% lethal concentration (LC_50_) of 6.466 × 10^4^ and 4.747 × 10^4^ spores/mL, respectively, and the 50% lethal time (LT_50_) was 2.89 and 3.54 days at an LC_50_ of 1.0 × 10^7^ spores/mL, respectively (Ying and Feng, 2019). This strain has the characteristics of high efficiency and rapid insecticidal action, and it therefore holds great potential for development as a fungal biological pesticide. Kaur et al. (2019) found that *Alternaria destruens* α-glucosidase inhibitors can cause high mortality in *Spodoptera litura*, delay its development, affect adult emergence, and induce adult malformation. Nutritional analysis showed that the α-glucosidase inhibitors were toxic and had antifeedant activity for various food utilization parameters of *S. litura*, and they also inhibited the activity of *S. litura* digestive enzymes (Kaur et al., 2019). Based on a network analysis of potential microbe combinations, Toju and Tanaka (2019) found that several fungal groups (*Orbiliales, Rhizopus*, and *Hypocreales*) may prey on nematodes, and *Pochonia* (Hypocreales) and *Purocillium* (Hypocreales) coexist in the soybean rhizosphere on a small spatial scale (Degenkolb and Vilcinskas, 2016). After coming into contact with nematodes, *Purpureocillium lilacinum* produces a large number of thickened hyphae, which can penetrate the eggshell, larva, or adult body of nematodes and subsequently absorb nutrients and reproduce, destroying the normal physiological metabolism of the egg, larva, or adult and resulting in nematode death (Toju and Tanaka, 2019). Future research must focus on the synergistic approach of insect-pathogenic microorganism preparations, such as adding foliar protective agents and slow-release agents to improve the survival rate of insect-pathogenic microorganisms and delay their validity period.

### Disease Control

With the increasing negative environmental and health effects of chemical fungicides and the increasing resistance of plant pathogens to these fungicides, the use of fungi as biological fungicides for the control of plant diseases has garnered attention from scientists worldwide (Jiang et al., 2019). The use of pathogenic fungi for controlling plant fungal diseases has broad and significant developmental prospects for establishing environmentally friendly ecological agriculture, improving the quality of agricultural products, and developing sustainable agriculture strategies. The consensus among researchers is that there are nine species of *Trichoderma*: *T. harzianum, T. viride, T. polysporum, T. koningii, T. lamatum, T. lignin, T. reesei*, and *T. hametum*, five of which have biocontrol potential. At present, the most commonly used species for this purpose is *T. harzianum*, which is currently used for the biocontrol engineering of bacteria (Filizola et al., 2019). Under greenhouse conditions, *T. harzianum* T82 and *Trichoderma* strain NF9 bran culture (10^7^ CFU/g) can be used to treat the soil (dosage: 0.6%, W/W), as they are effective against *Sclerotium rolfsii* Sacc., *Rhizoctonia solani*, and *Pythium aphanidermatum*. The inhibition rates of these two *Trichoderma* strains against the three species were 46.5, 28.4, and 81.2%, respectively (Filizola et al., 2019). *T. asperellum* T8a has an inhibitory effect on *C. gloeosporioides in vitro* and *in vivo*. *T. pseudocone* SMF2 can significantly reduce the severity of soft rot disease caused by *Erwinia arotovora* spp. in Chinese cabbage petioles and significantly increase the fresh weight of the ground and root system and the root-to-shoot ratio, resulting in a growth-promoting effect. Mechanisms in *Trichoderma* that are antagonistic to plant pathogens include heavy parasitism, antibiosis, and competition (Costa et al., 2019; Zhao et al., 2020). The application of phytopathogenic fungi in the field of biological control has shown great developmental prospects, but if they are to become an important disease management tool for crops, a large number of reliable and efficient biological agents must be produced (Antweiler et al., 2017; Garrigues et al., 2018; Loc et al., 2019; Vitorino et al., 2020). *Trichoderma* produce a variety of cell wall-degrading enzymes during the parasitism process to dissolve the cell walls of pathogenic fungi and achieve biocontrol effects. A protease produced by *T. harzianum* can degrade the pathogenic fungi that digest the plant cell wall, directly inhibit their germination, inactivate their enzymes, and prevent them from invading the plant cells (Berg et al., 2005; Pereira et al., 2014; Zhang et al., 2018; Abro et al., 2019). Endophytic fungi and pathogenic fungi can be converted into each other, and the conversion of endophytic fungi to plant pathogenic fungi may be related to the use of hybrid crop strains and the reduction in genetic diversity of crops. *T. harzianum* can rapidly grow in the roots and leaves of plants. By occupying sites on the plant surface, *T. harzianum* forms a protective cover; in this way it prevents pathogenic fungi from contacting the root system or leaf surface of the plant, thereby providing protection and ensuring the healthy growth of plants (Stopnisek et al., 2016; Panstruga and Kuhn, 2019; Yakti et al., 2019). However, it is still unclear whether biocontrol fungal preparations can successfully colonize the surface of plant roots and whether these colonized fungi are beneficial for plant growth. There are still many problems to be solved in the application of *Trichoderma* for the biological control of plant diseases. The first is the need to discover strains with strong adaptability and stable biocontrol effects. At the same time, it is necessary to improve the processing level of *Trichoderma* inoculants, extend the shelf life of biocontrol inoculants, and produce highly efficient and stable biological agents.

### Grass Control

Toxins secreted by many plant-pathogenic fungi exhibit herbicidal activity. Microbial herbicides developed using microbial metabolites, particularly phytopathogenic toxins, usually have multiple target sites, complicating the development of resistance in weeds (Zhang et al., 2011). Microbial herbicides have specificity, a high success rate for development, and are easy to process. This is one of the hotspots in herbicide research, as microbial herbicides present good application prospects. For example, cytosporaphenone C of *Cytospora rhizophorae* A761 exhibits good herbicidal activity; the inhibition rate of cytosporaphenone C on the radical growth of *Echinochloa crusgalli* and *Amaranthus retroflexus* was 94.6 and 77.3%, respectively (Liu et al., 2007). The unique mode of action of phytopathogenic toxins with herbicidal activity, which is completely different from the mode of action of chemically synthesized herbicides, makes these toxins extremely attractive as potential new herbicides (Liu et al., 2007). For example, moniliformin is a toxin produced by *Fusarium moniliforme*. It is phytotoxic and prevents the mitosis of corn root meristem cells in the middle stage of division (Reichert et al., 2019). Cercosporin is a red toxin that was first isolated from several species of the fungus *Cercospora* in 1950. This photodynamic pigment is a strong photosensitizer. In the presence of both light and oxygen, it generates singlet oxygen and superoxide ion free radicals, resulting in rapid cell membrane peroxidation and cell death. Toxins of pathogenic fungi have the potential to produce herbicidal active substances, but at present, they are mainly studied for identification and determination of pathogenicity. There have been few studies on the herbicidal activity of the secondary metabolites of pathogenic fungi. However, it is known that 2, 5-Anhydro-D-glucoside is produced with mild phytotoxicity by the phytopathogenic fungus *Fusarium solani* in *Senna obtusifolia, Abutilon theophrasti*, and some species of morning glory. Additionally, macrocidins (A and B) obtained from the fungus *Phoma macrostoma*, when used as a biocontrol to infect *Cirsium arvense*, are cyclic tertiary acids. This new type of compound has great potential as a template for designing new herbicides (Reichert et al., 2019). Qiang et al., 2010 found that the AAC-toxin produced by *Alternaria alternata* and isolated from diseased plants of the malignant weed *Ageratina adenophora* has herbicidal activity. It can control a broad-spectrum of gramineous and broad leaf weeds, and it has a fast killing rate, similar to that of paraquat. Zhang et al. (2011) showed that when barnyardgrass was treated with the onion purple blotch toxin, it inhibited the growth of barnyardgrass. However, the application of plant-pathogenic mycotoxins is still in the laboratory research stage. The development of bioengineering technologies, such as cell engineering and tissue culturing, is expected to allow the rapid development of practical applications of natural phytopathogenic fungal toxins. In order to reduce the resistance of pest to chemical pesticides and transgenic maize as soon as possible and protect the ecological environment, it is necessary to further increase the investment to provide a reliable guarantee for the research and development of fungal insecticides and related application technology.

### Relief Continuous Cropping Obstacles

Arbuscular mycorrhizal (AM) fungi are beneficial to the terrestrial ecosystem; they can establish symbiotic relationships with more than 80% of higher vascular plants to form a specific “mycorrhizal” structure (Evelin and Kapoor, 2014). AM fungi can expand the absorption area of the host plant's roots, promote the plant's absorption of soil nutrients, enhance its resistance to abiotic stress, and improve its disease resistance (Hodge and Fitter, 2010). AM fungi are the most abundant and beneficial fungi in the soil, and they can reduce populations of *Phytophthora parasitica, Rhizoctonia solani, Fusarium solani*, and *Ralstonia solanacearum* (Whiteside et al., 2012). Inoculation with AM fungi can reduce the root damage caused by pathogens and significantly increase the content of secondary metabolites in the root system, thereby inhibiting pathogen reproduction (Laparre et al., 2014). AM fungi can also induce the production of defensive enzymes in plants, helping to reduce potential pathogen damage. The development of AM fungi can effectively improve soil organic matter content, soil grain structure, and air porosity. The AM hyphae penetrate through the tiny pores between soil particles. Their secretions, including saccharin and organic acids, can be used as adsorbents for adhesion between soil particles, which promotes the formation of soil aggregates, improves soil pH, and enhances the activities of enzymes such as urease, invertase, and catalase, thereby reducing the damage incurred by continuous cropping (Whiteside et al., 2012). Upon the symbiotic association of AM fungi with the host plant, the mycelium will quickly occupy the corresponding niche and will inevitably compete with the pathogen and reduce its infection site. At the same time, when pathogenic fungi infect mycorrhizal plants, the AM fungi will induce proline glycoprotein production in the root system of the host plant to immediately initiate a rapid defense response, increase the strength of the cell wall, hinder the invasion of pathogenic fungi into the root system, and reduce the root-infection rate (Laparre et al., 2014). AM fungi can also synergize with other beneficial microorganisms in the soil, stimulate the activity of microorganisms that have antagonistic effects on soil-borne pathogens, increase the number of beneficial microorganisms, reduce the number of pathogens, and enhance the plant's resistance. After inoculation with AM fungi, the expression of defense genes, such as *PAL5* and the disease-related protein chitinase gene *ChibI*, is regulated at the transcriptional level, inducing the synthesis of disease-related proteins and increasing the crop's resistance to disease (Evelin and Kapoor, 2014). Although some speculate that endophytic fungi may use secondary metabolites to regulate the balance between fungi and the balance between fungi and bacteria, this speculation is still preliminary and requires more in-depth research.

## Conclusions and Prospects

In-depth research on diverse plant-pathogenic fungal species is the basis for understanding plant diseases, and it can provide basic information and a scientific basis for further in-depth studies on pathogenic mechanisms, disease infection cycles, and effective pest control (Wang and Coleman, 2019). However, for the major interactions between fungi and hosts, there is still a lack of systematic research on signaling identification and defense-response activation processes, and little is known about the disease-resistance characteristics and mechanisms of major crops (Wiemann et al., 2013). Fungal diseases of fruits not only harm the fruit in the field and reduce yield, but also result in huge losses of commercial value after harvest due to characteristics of latent infection (Sossah et al., 2019). However, there are few studies on the pathogenic mechanisms underlying these diseases. In addition, studies on mixed infections and synergistic pathogenicity of multiple pathogenic fungi are rare. The following questions remain unresolved in the field of plant-pathogenic fungi and their toxins: How can plants restrict pathogenic microorganisms while interacting with beneficial ones? How do environmental factors affect plant-microbe interactions? How can basic research on plant immunity be applied to crop resistance of pests and diseases? How do microbe–microbe interactions affect plant–microbe interactions? Why do some pathogenic microorganisms require many effector proteins, whereas others need few? How do pathogenic fungi develop new toxic functions? In the future, scholars will strive to answer these major scientific questions and seek breakthroughs in the basic theory of plant immunity and in the improvement of crop resistance.

In summary, to effectively control the occurrence of and harm from fungal diseases in plants, research needs to be continued on a number of related topics. Identification of characteristics and signaling pathways, regulation of defense-response activation processes, and isolation and functional verification of pathogenic fungal effectors will surely lay a theoretical foundation for the development of new approaches for controlling pathogen damage and selecting resistant varieties;. Currently, the disease-resistant genes are isolated and identified using traditional molecular genetic methods, such as breeding and single-gene identification and cloning, which are inefficient. An important characteristic of plant disease-resistance genes is their huge diversity. In the future, more attention needs to be paid to the genome of disease-resistant plant and genome-wide association studies. With the efficient identification of disease-resistance genes, their diversity can be fully utilized for the breeding of plants for disease resistance. Finally, a combination of genomics, transcriptomics, proteomics, and metabolomics will help to better identify new metabolites and metabolic pathways in plant-pathogen interactions, and help us to understand the mechanisms underlying plant responses to pathogenic fungal stress at the whole-organism level.

## Author Contributions

JR and BX designed the review. YP and SL collected the data and drafted the manuscript. JY and JC contributed to drawing the plots. YT and AG prepared the manuscript and contributed to manuscript revision. YP, XY, and JR critically revised the manuscript and approved the final version. All authors contributed to the article and approved the submitted version.

## Conflict of Interest

The authors declare that the research was conducted in the absence of any commercial or financial relationships that could be construed as a potential conflict of interest.
